# Congenitally Corrected Transposition of the Great Arteries in the Adult

**DOI:** 10.21470/1678-9741-2021-0528

**Published:** 2022

**Authors:** Fernando Amaral, Anne Marie Valente, Paulo Henrique Manso, Luiz Gustavo Gali, Maria Fernanda Braggion-Santos, Julia Mignot Rocha, Walter Vilella de Andrade Vicente, André Schmidt

**Affiliations:** 1 Adult Congenital Heart Disease Unit, Hospital das Clínicas, Facultdade de Medicina de Ribeirão Preto, Universidade de São Paulo, Ribeirão Preto, São Paulo, Brazil; 2 Cardiology, Boston Children’s Hospital, Boston, Massachusetts, United States of America; 3 Pediatrics, Hospital das Clínicas, Faculdade de Medicina de Ribeirão Preto, Universidade de São Paulo, Ribeirão Preto, São Paulo, Brazil; 4 Cardiology, Hospital das Clínicas, Faculdade de Medicina de Ribeirão Preto, Universidade de São Paulo, Ribeirão Preto, São Paulo, Brazil; 5 Cardiothoracic Surgery, Hospital das Clínicas, Faculdade de Medicina de Ribeirão Preto, Universidade de São Paulo, Ribeirão Preto, São Paulo, Brazil; 6 Cardiology, Faculdade de Medicina de Ribeirão Preto, Universidade de São Paulo, Ribeirão Preto, São Paulo, Brazil

**Keywords:** Congenitally Corrected Transposition of The Great Arteries, Systemic Right Ventricle, Adult, Heart Failure, Congenital Heart Defects.

## Abstract

**Introduction:**

Congenitally corrected transposition of the great arteries (CCTGA) is a rare anomaly. Current data available regarding adult cases is derived from small series, information simultaneously presented in pediatric publications, and one classical multicenter study. This review, not aimed to exhaust the subject, has the purpose to examine the literature addressing presentation, diagnostic methodology, and management of afflicted adult patients.

**Methods:**

A comprehensive search was undertaken in three major databases (PubMed, Cochrane, SciELO), using the keywords “congenitally corrected transposition of the great arteries” and “adults”. Relevant articles in English, Spanish, and Portuguese were extracted and critically appraised in this review. Steps for study selection were: (1) identification of titles of records through databases searching, (2) removal of duplicates, (3) screening and selection of abstracts, (4) final inclusion in the study.

**Results:**

Four hundred sixty-five publications on CCTGA in adult patients were retrieved, and 166 were excluded; 299 studies were used for this review including 76 full-text articles, 70 studies related to general aspects of the subject, and, due to the small number of publications, 153 case reports. Sixty-one articles referring to combined experiences in pediatric and adult patients and judged to be relevant, but retrieved from another sources, were also included.

**Conclusion:**

Albeit clinical presentation and diagnostic criteria have been well stablished, there seems to be room for discussion related to clinical and surgical management of CCTGA in adults. Considering the rarity of the disease, well designed multicenter studies may provide answers.

**Table:** 

Abbreviations, Acronyms & Symbols				
AC	= Anatomic correction		NYHA	= New York Heart Association
ACHD	= Adult congenital heart disease		PA	= Pulmonary artery
AO	= Aorta		PC	= Physiological correction
ASD	= Atrial septal defect		PS	= Pulmonary stenosis
AV	= Aortic valve		PV	= Pulmonary valve
CCTGA	= Congenitally corrected transposition of the great arteries		RA	= Right atrium
CHD	= Congenital heart defects		RCA	= Right coronary artery
CMR	= Cardiac magnetic resonance		RV	= Right ventricle or right ventricular
LA	= Left atrium		TR	= Tricuspid regurgitation
LV	= Left ventricle or left ventricular		VSD	= Ventricular septal defect

## INTRODUCTION

Described in 1875 by Von Rokitansky, an Austrian pathologist, congenitally corrected transposition of the great arteries (CCTGA) is an intriguing cardiac malformation representing approximately 0.5% of all congenital heart defects (CHD)^[1^,^2]^. Publications in the last several decades have addressed many aspects of this entity in children, particularly concerning its management. Information regarding adult patients is scarce, which is possibly related to the small number of individuals registered in tertiary centers. As children grow, and diagnosis and treatment improve, an increased number of patients are expected to reach adulthood and require follow-up in specialized units. This review, which is not an attempt to exhaust the subject, highlights some of the main diagnostic features in adults with CCTGA and discuss the available management options illustrated by some cases from our practice.

## MORPHOLOGY

Characterized by a combination of atrioventricular and ventriculoarterial discordance, CCTGA has a wide spectrum of possible associated morphologic features and clinical profiles. The morphologic mitral valve and left ventricle (LV) receive the systemic venous blood and are connected to the pulmonary artery (PA) while the morphologic right ventricle (RV) and tricuspid valve receive the pulmonary venous blood and are connected to the aorta, which is anterior and leftward of the PA^[2^,^3]^. *Situs solitus* is the rule, the apex of the heart remains usually leftward but the cardiac position is frequently more mesocardic than usual^[2]^. Dextrocardia occurs in about 20% of the cases^[4]^.

More than 90% of these patients have intracardiac anomalies, which may influence the age at clinical presentation and adult survival, depending on the specific anatomic abnormalities and their physiologic significance^[2]^. One study looking at autopsies of patients with CCTGA noted an *abnormal tricuspid valve* in 94% of the cases; some of these valves were reported to be anatomically similar to Ebstein’s anomaly^[3]^. Progressive regurgitation is a well-recognized complication, the severity of which has been reported to be an important prognostic factor for these patients. A *ventricular septal defect (VSD)* occurs in 60 to 80% of the cases, is frequently large, and is usually perimembranous. *Valvar* or *subvalvar pulmonary stenosis (PS)* affects 50% of the patients, often associated with a VSD. *An atrial septal defect (ASD)*, either isolated or associated with other defects, is less frequent. The *coronary arteries and veins* are typically abnormal with the morphological left coronary artery arising from the patient’s right-sided aortic sinus and the morphological right coronary artery (RCA) from the left-sided sinus with concordant ventricular supply. Single sinus origin of the two arteries, arterial hypoplasia, and course variations may occur, making preoperative delineation mandatory^[5]^. The *conduction system* is abnormal, including the anteriorly situated atrioventricular node^[6]^. The conducting tissues descend from this node and, when a VSD is present, they are related to its anterior and inferior borders, which has significant implications when surgery is considered. Also, pathological changes in older patients may predispose them to arrhythmias and heart block. *RV dysfunction* affects many patients and is reported to be associated with an increased distribution of highly sensitive troponin T, and the extent of this increase is noted to have important prognostic implications^[7]^. This has been described as a multifactorial process probably secondary to continuous exposure to systemic pressure/resistance and possibly inadequate RV myocardial perfusion despite concordant RCA-RV anatomy^[8]^.

## PRESENTATION AND DIAGNOSIS

Adults with CCTGA may remain undiagnosed up to the ninth decade, particularly those without associated defects^[9^-^11]^. Considering this, it is very likely that some people with CCTGA are never diagnosed so we cannot know its true incidence. Based on the current data, life expectancy is significantly reduced compared to the general population, and the majority of patients progress to cardiac failure by the fourth or fifth decade of life^[12]^. Thus, early clinical recognition is crucial to prevent adverse consequences. Other forms of presentation include arrhythmias, abnormal cardiovascular signs like dyspnea, palpitations, murmurs, and an abnormal electrocardiogram or chest radiography^[13^,^14]^.

## BEDSIDE FINDINGS

**VSD/PS:** Patients with these lesions are only occasionally first diagnosed during adult life, since the murmur is quite obvious and detection early in life is possible. Balanced lesions may lead to minimal symptoms, and cyanosis and diagnosis will be late, specially if access to medical care is limited. When significant PS occurs, severe cyanosis with limited exercise tolerance is the rule. The systolic *VSD murmur* is maximal along the mid to lower left sternal border; due to the abnormal position of the pulmonary valve, the systolic *PS murmur* is best heard inferior or to the right of its expected site.

**Tricuspid regurgitation (TR):** Although TR occurs quite frequently and typically worsens with time, its severity may vary^[3]^. The combination of moderate to severe lesions and RV dysfunction may lead to heart failure symptoms^[11]^. Concomitant PS, usually associated with a VSD, may reduce the degree of TR which can otherwise be exacerbated by intracardiac surgery^[15^,^16]^. The severity of PS and the amount of TR may affect RV remodelling with respect to geometry, hypertrophy, and systolic and diastolic function, which would also impact surgical management. Due to the abnormal plane of the ventricular septum, the morphologic RV is closer to the left sternal edge and the *TR systolic murmur* is best heard near the left lower sternal border. Its appearance late in life is related to progressive valve regurgitation.

Independently of the associated lesion, a *single second sound* is an important physical sign, commonly heard at the left upper sternal border and caused by the anterior position of the aortic valve in relation to the pulmonary valve. Loudness may vary depending on the degree of the aortic valve anteriorization and may be somewhat masked when a harsh murmur is present. Its identification since birth, particularly in young patients without associated lesions and soft or absent murmurs, should draw attention for its cause and eventually lead to an early diagnosis of CCTGA.

**Fig. 1:**
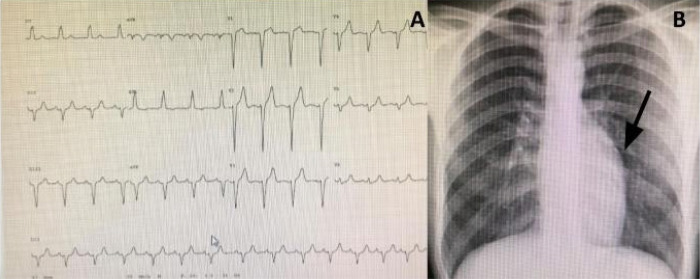
Typical electrocardiogram (A) and chest X-ray (B) of an asymptomatic 29-year-old patient with congenitally corrected transposition of the great arteries plus mild tricuspid regurgitation (arrow=humped appearance).

**Electrocardiogram:** Frequent findings include a q wave in V1 and inferior leads with absent q wave in V6 (Figure 1A). However, this is not the case for all patients. Complete heart block is also seen, may be found incidentally or in symptomatic patients, and may be the first clue to diagnosis^[13]^.

**Chest radiography:** The so-called “humped appearance” of the left border (Figure 1B, arrow), is common but not always present. Variations in heart shape can be found, depending on the presence and severity of the associated lesions as well as on the degree of heart dysfunction^[17]^.

**Echocardiography:** The peculiar discordant atrioventricular and ventriculoarterial connections and the abnormal spatial relationship of the great arteries are usually seen, sometimes as the first diagnostic clue. The initial segment of the great vessels run in parallel, and the ventricular arrangement is more often side-by-side or superior-inferior rather than left/right, anterior/posterior as found normally. The VSD is usually perimembranous, and its precise features are important when surgical intervention is considered. It is also important to determine the location, extent, and type of valvar and/or subvalvar PS (fibrous diaphragm, membranous septum aneurysm, or accessory mitral tissue). In some patients, TR is noted to arise from inferiorly displaced tricuspid valve leaflets, anatomically similar to Ebstein’s anomaly. Routine follow-up of these patients is important as the TR in this setting tends to progress and can be associated to RV dysfunction.

**Fig. 2:**
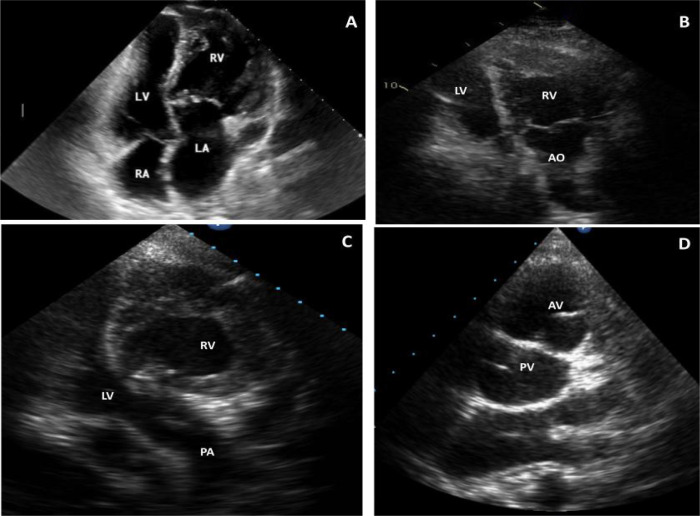
Bidimensional echocardiograms in patients with congenitally corrected transposition of the great arteries. A) 16-year-old patient, four-chamber view. Discordant atrioventricular connections plus moderate tricuspid regurgitation (TR) and mild pulmonary stenosis (PS); B) 35-year-old patient, apical view. Left-sided right ventricle (RV) connected to aorta (AO) plus mild TR/PS after Rastelli operation; C and D) 24-year-old patient with mild TR. Apical view (right-sided left ventricle [LV] connected to pulmonary artery [PA]) (C) and short axis parasternal view (aortic valve [AV] anterior and to the left of pulmonary valve [PV]) (D). LA=left atrium; RA=right atrium.

RV function assessment is crucial. Late presentation with pre-existing RV dysfunction has been reported to impact survival. Echocardiographic assessment might be challenging, particularly due to the complex morphologic features of the RV cavity, including coarse trabeculations in the medial and apical regions. However, reliable echocardiographic parameters do exist including tricuspid annular plane systolic excursion (or TAPSE), fractional area changing, tricuspid ring tissue doppler S’ wave, myocardial performance Tei index, and global longitudinal strain by the speckle tracking method^[18]^. Recent studies have highlighted the benefits of assessing the longitudinal functional parameters, and the global longitudinal strain has been reported to be a highly sensitive marker of RV dysfunction in patients with RV ejection fraction < 45%^[19^-^21]^(Figure 2).

**Fig. 3:**
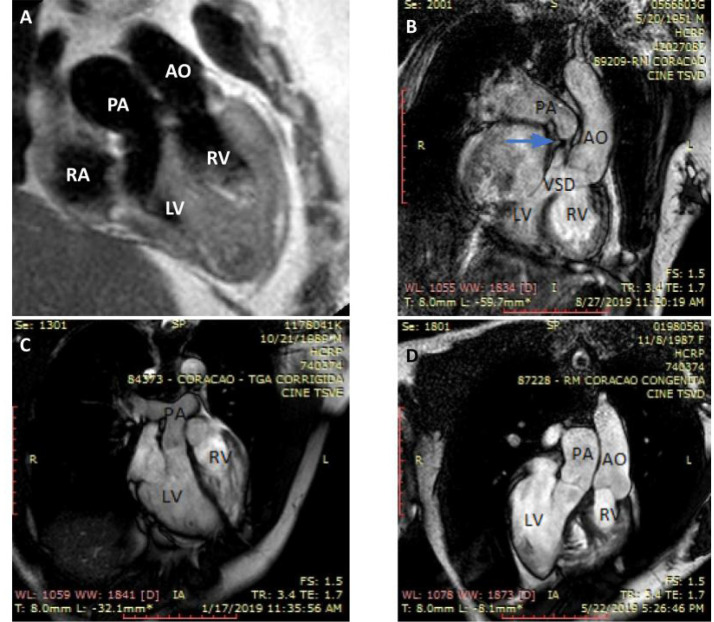
Magnetic resonance aspects of four patients with congenitally corrected transposition of the great arteries. A) Mildly symptomatic 50-year-old patient with moderate-severe tricuspid regurgitation (TR) showing discordant right-sided atrioventricular connection and bilateral discordant ventriculoarterial connections; B) symptomatic 69-year-old patient with large ventricular septal defect (VSD) plus severe pulmonary stenosis (blue arrow); C) asymptomatic 29-year-old patient with mild TR; D) 31-year-old patient with a mesocardiac heart after atrial septal defect closure and tricuspid valve replacement (same patient of Figure 7). AO=aorta; LV=left ventricle; PA=pulmonary artery; RA=right atrium; RV=right ventricle.

**Cardiac magnetic resonance (CMR):** Albeit not uniformly available, it is considered the gold standard imaging modality. Besides providing super imaging quality for vessels emerging from the heart, excellent anatomic detailing including ventricular volumes measurements and quantification of shunt and valvar regurgitation can be obtained (Figure 3). Despite of the complex morphology, cine images in short axis allow for precise RV function calculation based on systolic and diastolic dimensions^[2^,^22]^. Meticulous delineation of the RV contour outside of the trabeculations is necessary to make the method more reproducible and to determine the RV pattern of contraction. This is important since dyssynchronous RV free-wall motion in the setting of dyssynchronous ventriculo-ventricular interaction has a significant impact on cardiac output and major cardiac events have been reported even in patients who are mildly symptomatic^[23^,^24]^. Myocardial fibrosis can be detected by delayed enhancement techniques using gadolinium as contrast agent and it has been reported to be associated to progressive clinical deterioration, arrhythmia, poor exercise tolerance, and RV dysfunction^[24]^. Due to its high cost, CMR is not available in many centers, and, consequently, an ideal follow-up strategy might be lacking for a good number of adults who need sequential RV function evaluation for intervention planning. In paced patients, echocardiography or radionuclide ventriculography should be used.

**Cardiopulmonary exercise test:** This functional investigation has been increasingly used in adult congenital heart disease (ACHD) patients to identify those with a potential for an unfavorable outcome and to inform decisions regarding the need for timing and type of intervention^[25]^. As in other complex CHD, a diminished aerobic capacity occurs in adults with CCTGA, suggestive of a diminished cardiac function^[24^,^25]^. A significant correlation was also found between the predicted peak oxygen uptake (%pVO2) and RV ejection fraction by CMR and the Tei index obtained by echocardiography^[26]^. A recent study involving adults with a systemic RV showed that peak oxygen uptake, peak heart rate, and percentage of maximal heart rate with exercise were significantly lower when compared to a control group. In that study, reduced exercise capacity was associated with impaired systemic RV function, severe TR, and chronotropic incompetence^[27]^. Exercise testing should be done routinely as it provides objective data from which to assess for evidence and extent of clinical deterioration as well as the rate of progression. Training may improve exercise capacity, and patients not considered to be at significant risk for arrhythmias or sudden death with exercise should be encouraged to engage in regular physical activity.

**Cardiac catheterization:** Although the non-invasive investigation can usually establish the diagnosis, cardiac catheterization has its role, mainly for preoperative evaluation when pulmonary vascular resistance and PA morphology need to be determined and, occasionally, to assess for presence of associated lesions. Also, selective coronary arteriography is important since abnormal morphology and acquired lesions may occur, which can be crucial for medical management and surgical planning.

## MANAGEMENT

**Fig. 4:**
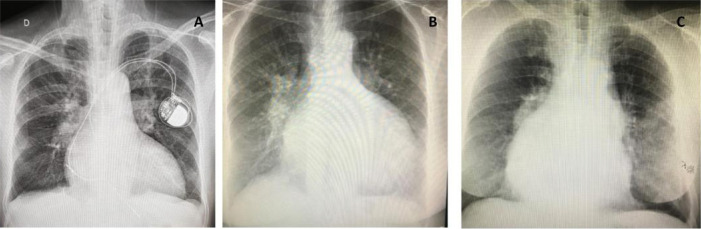
Chest radiographs of three patients with congenitally corrected transposition of the great arteries in heart failure. A) Deceased 70-year-old patient with tricuspid regurgitation (TR) + right ventricular (RV) dysfunction; B) 73-year-old patient with moderate TR/RV dysfunction (previous atrial septal defect closure) in NYHA class II; C) 69-year-old patient who refused surgery with large ventricular septal defect + severe pulmonary stenosis in NYHA class II (same patient of Figure 3B). NYHA=New York Heart Association.

**Medical treatment:** Patients with isolated CCTGA, having associated lesions of minimal or no clinical significance, may enjoy an almost normal life as long as RV function is preserved. Reports of older patients having normally functioning RV provide evidence that, in some cases, the RV is able to adapt remarkably well to systemic pressure^[28]^. Predicting who will develop heart failure secondary to RV dysfunction is difficult, but symptoms usually start after the 4^th^ decade of life, sometimes with associated LV dysfunction^[12]^ (Figure 4). Why some patients develop RV dysfunction early in life and others do not is not entirely clear and is, likely, multifactorial. Patients with CCTGA that have a single RCA supplying the morphological, hypertrophied RV are at risk for RV ischemia, which may be complicated and/or exacerbated by coronary artery disease^[21]^. An experimental model of RV hypertrophy induced by PA banding in rats six hours after birth was created^[29]^. Different of another model in which young larger animals were used^[30]^, this recently reported project aimed to study the pathophysiological changes in CHD with increased afterload which could, eventually, be applied to patients. For medical treatment, reduction of afterload with angiotensin-converting enzyme inhibitors or angiotensin II receptors might help patients with RV dysfunction despite limited data available. Though there have not been any studies to guide clinicians, the empiric use of diuretics, including aldosterone antagonists, essential for symptomatic patients with LV failure, are commonly used in patients with systemic RV failure. Beta blockers should be used with caution due to the propensity for heart block^[14]^.

The RV dysfunction is typically progressive, and patients may report symptoms attributable to heart failure. For some patients, dyssynchronous ventriculo-ventricular interactions contribute significantly, and contraction and relaxation are suboptimal. Some patients may benefit from placement of an atrio-biventricular pacing system by a cardiac electrophysiologist trained and experienced performing the procedure in patients with complex CHD. It is essential to have detailed analysis of the coronary sinus and coronary veins, including a selective venogram^[31]^. Surpassing the eventual anatomic barriers, this supportive therapy, if used in addition to conventional therapy, can improve symptoms^[32^,^33]^. Medically resistant heart failure may unfortunately afflict patients. Mechanical support, not necessarily used as a bridge to transplantation, should be available, and several types of devices might be used. Numbers are small, but according to the Interagency Registry for Mechanically Assisted Circulatory Support, CCTGA is a frequent cause of device need in adults with CHD^[34]^. Tertiary expertise and an adequate procedure planning, which should include patient awareness of possible complications, are required. Collaborative multicenter studies seem to be necessary to improve knowledge and benefit patients^[35]^.

**Pregnancy:** In two studies, 32 patients aged 18 to 40 years who had 65 pregnancies had their experience reported in order to define maternal and neonatal outcomes^[36^,^37]^. Despite the small sample size, pregnancy was successful in most of them. However, supraventricular arrhythmias and RV dysfunction occurred in a few patients. Miscarriages and elective termination of pregnancy were uncommon, and CHD in offspring was rare. Preconception counseling is important, and prenatal care should be done at a tertiary center. Given the physiological changes that occur during pregnancy, risk for maternal cardiac complications increases significantly in those patients who have RV dysfunction and/or more than moderate TR that predates their pregnancy.

**Arrhythmias:** Physicians caring for these patients need to be aware of the potential for arrhythmias, including those leading to sudden death, which can occur in patients with CCTGA, even in those without severe RV dysfunction. Recently, heart block was detected in 14 (36%) of 39 patients, most of them of 3rd degree necessitating atrio-biventricular pacing placement in order to avoid ventricular desynchrony^[38]^. Routine Holter monitoring is very important for these patients, as there is a 2% risk annually to develop heart block spontaneously, most often in patients with additional cardiac anomalies^[2^,^10^,^12^,^13]^. It should be remembered that complete heart block may be acquired after surgical VSD closure. Atrial flutter/fibrillation may also appear in these patients, either precipitating or exacerbating RV dysfunction, and require treatment^[12]^. Implantable cardioverter defibrillator represents the first-line therapy for secondary prevention of sudden cardiac death in patients with repaired or unrepaired associated lesions, particularly when RV ejection fraction < 35% and other additional risk factors are present^[39]^.

**Surgical treatment:** There are only a few reports that specifically address surgery for CCTGA in adults^[14^,^40^-^43]^. There is not a single approach that would be appropriate for all patients since the disease is rare and the specific anatomy and physiologic manifestations are highly variable. Most of the repairs are individually tailored, including both biventricular (physiological or anatomical) and univentricular repair.

**Physiological correction (PC):** In this approach, the abnormal ventriculoarterial connections are left intact. The most frequently performed operation is the *tricuspid valve replacement* in cases with more than moderate TR, either as an isolated procedure or at the time of intracardiac repair of other lesions^[11^,^14]^. The regurgitation is usually progressive, may lead to heart failure symptoms, valve repair is not advisable, and a mechanical or a bioprosthetic valve are the options available^[14^,^15^,^40^,^42^,^43]^. The interplay between the RV dysfunction and TR severity (which comes first?) is not yet clear; likely there are patients in whom the TR is native and results in a volume loaded RV with subsequent RV failure and dilation that exacerbates the TR. For some other patients, the RV dysfunction may occur first, causing RV dilatation and progressive TR, which then leads to a volume load worsening RV function and dilatation with further exacerbation of the TR. RV dysfunction increases the surgical risk and may be preventable by an early operation^[12^,^14^,^15^,^44]^. Close follow-up is advisable with periodic evaluation of RV function since low mortality during valve replacement can be accomplished if ejection fraction is > 40%^[44]^. Good results are expected, and the risk factors for mortality or transplantation late after valve replacement included systemic RV fraction < 40%, atrial fibrillation, and nonsystemic ventricular systolic pressure > 50 mm/hg at the time of operation^[42^,^43^,^45]^.

**Fig. 5:**
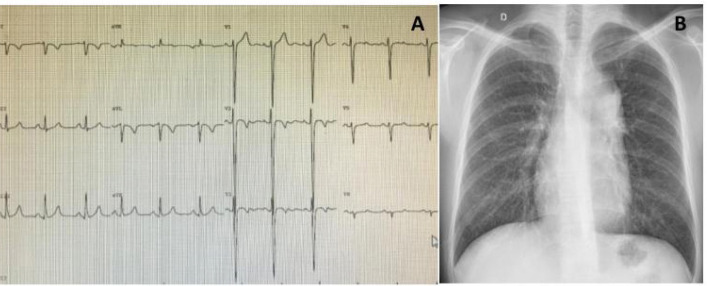
Atypical electrocardiogram (A) and chest radiography (B) of a 34-year-old cyanotic patient with congenitally corrected transposition of the great arteries + ventricular septal defect + severe pulmonary stenosis who refused treatment.

Some patients might need *closure of a large VSD*, usually during the same operation as that as in which pulmonary outflow obstruction is being addressed. These combined procedures are not recommended in asymptomatic patients with balanced situation and not severe lesions and they are not a common practice in adults, since patients with severe lesions are usually treated during pediatric age. However, a challenging situation might happen, particularly in very cyanotic patients^[46]^ (Figure 5). Due to its peculiar morphologic features, the VSD closure may cause heart block. Despite advanced techniques, that still occurs even in the most proficient surgeons’ hands^[47]^. One interesting aspect is the protective effect of these lesions on the tricuspid valve functioning and, on the contrary, the TR worsening reported after VSD closure with or without PS relief due to a leftward shift of the interventricular septum after operation^[15^,^16^,^48^,^49]^.

**Fig. 6:**
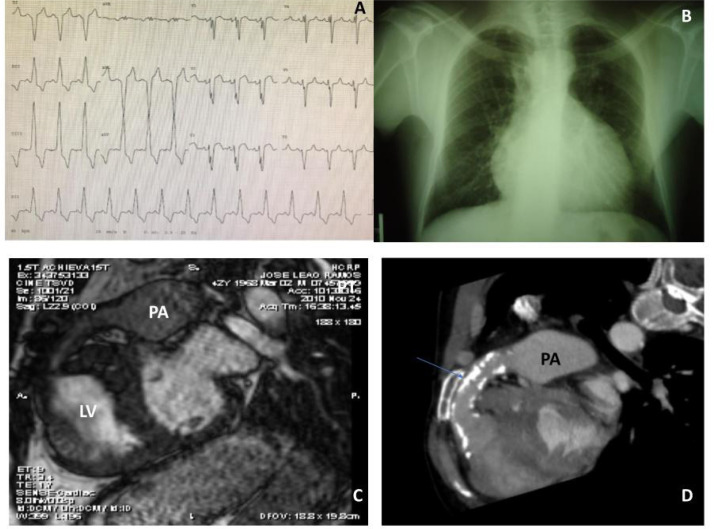
Preoperative electrocardiogram (A), chest radiography (B), magnetic resonance (C), and computed tomography (D) of a patient with congenitally corrected transposition of the great arteries who had a Rastelli operation at age 12 and died immediately after a homograft replacement for a severely calcified and stenotic valve (arrow) at age 40. LV=left ventricle; PA= pulmonary artery.

Patients treated at any age by a Rastelli operation might need a *conduit replacement* during adult life (Figure 6).

**Fig. 7:**
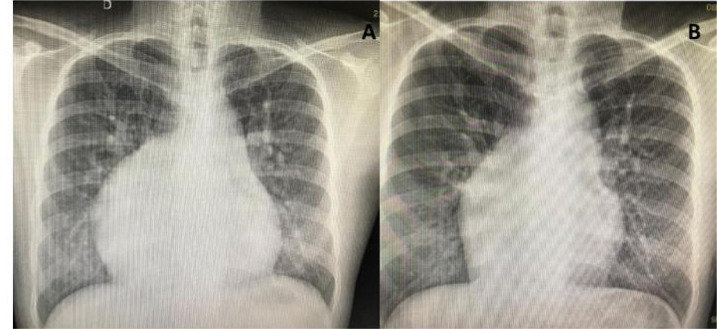
Preoperative (A) and three-year postoperative (B) chest radiographs in a currently well 31-year-old patient with congenitally corrected transposition of the great arteries and a mesocardiac positioned heart submitted to a large atrial septal defect closure plus a mechanical tricuspid valve replacement (same patient of Figure 3D).

*Closure of a large ASD* may be necessary, either isolated or at the time of another defect correction (Figure 7).

*PA banding*, known to reduce TR severity in children, is still considered a controversial procedure^[50]^. Despite theoretically attractive, there are no reported experiences with this operation in adults.

The abovementioned operations have a common feature that is leaving the RV at systemic pressure. Experience with pediatric patients has shown that this strategy (PC) might be deleterious in the long term despite good early results^[49^,^50]^.

**Anatomic correction (AC):** Three decades have elapsed since Dr Ilbawi’s first description of the double switch operation for CCTGA in children, characterized as a Senning/Mustard operation plus either a Rastelli or Jatene operation^[51]^. Other procedures as the aortic, the double root, and pulmonary root translocation have also been suggested highlighting the basic principle that is to restore the morphologically LV to support the systemic circulation^[52^,^53]^. Recent analysis has stressed the need of surgical improvements for better late results. Several retrospective studies from different institutions have shown that the operation is feasible in children, particularly for those with a poor RV function and a normal, well-functioning LV at systemic pressure^[54]^. However, many of these patients have issues that arise in the long term. In a group of 113 patients operated on between 1991 to 2011 with a mean age of 3.2 years, atrioventricular and neo-aortic valve deterioration, Senning pathway obstruction, RV-PA conduit obstructions, and LV dysfunction have been documented, demanding long-term surveillance of these patients^[55]^.

In adults, AC has been occasionally reported, and the criteria for a safe patient selection has not yet been defined. Also, it is important to consider that the introduction of a new technique, attractive as it may be, should be approached with caution. An ethical dilemma, as well discussed as the adoption of the switch operation for classical transposition of the great arteries in children^[56]^, might influence the choice of the technique. Should we offer the patient a safer (physiologic) procedure or a procedure which may theoretically restore normal physiology (anatomic)? Although generally not considered an adequate option for adults with CCTGA, it is possible to verify that among several pediatric cases series, some patients over 15 years old treated by a double switch operation were included. We found that information interesting, which prompted us to do a more thorough search of the literature. Ultimately, we reviewed 35 references referring to the anatomical correction mentioned in a classic meta-analysis study and in another more recent publication^[57^,^58]^. Looking at these 35 articles, published between 1995 to 2018, we found that 19 of them reported surgical experience in children, and 16 others were related to children and adult cases. Although four of these publications included 106 to 189 patients, in 66% of the reports the number of patients were < 50 per article. Among the cases reported, we found that 16 of them were older than 15 years of age by looking at the methods of each article. An electronic message reply was obtained from three of these authors, from three different institutions, who confirmed the operation was done in five patients, four of them with good follow-up^[59^-^61]^. In 2013, Talwar, in a case series that included patients over 15 years of age, reported 15 patients operated on. Among them, five had a tricuspid valve operation, eight had a univentricular correction, and only one patient was submitted to AC (Senning/Rastelli), with no mortality at a mean follow-up of 49 months^[37]^. In 2016, Baruah reported performing a successful atrial switch plus a Rastelli operation in a 51-year-old patient with large VSD, severe PS, and severe TR. This patient, temporarily paced soon after surgery, had a good recovery, and was New York Heart Association class I at three months after operation^[62]^. Recently, Da Silva (personal communication) did a pulmonary root translocation plus a Senning procedure in a 46-year-old symptomatic patient with good RV function who had large VSD, moderate PS, and severe TR with good early follow-up. It should be emphasized that the abovementioned cases are small numbers and should not be used as evidence-based information to recommend this procedure for adults with CCTGA. However, they provide examples and demonstrate that these surgical approaches are feasible and seem to be appropriate for certain patients, which also reinforces the importance of making decisions on a case-by-case basis. Due to the CCTGA infrequent occurrence and anatomical variability, most surgical services have small number of adult cases, and a preferable surgical strategy has not yet been established. It seems reasonable and appropriate that each center develop clinical criteria based on which information is published and the individual center surgeon’s experience, training, and preference, in discussion with the cardiologists. Is it ethical to offer a double switch for an adult with CCTGA? Should data scarcity interfere in the patient-surgeon decision to go ahead with an apparently ideal procedure? However difficult to be accomplished, a multicenter study or a surgical consortium involving patients already operated on could possibly give us an idea about the benefits of this procedure in adults.

**Univentricular correction:** This technique can be a good option in cases with a hypoplastic RV, inadequate atrioventricular valves, and atypical VSD morphology. In a recent report of 15 patients operated on after the age of 15 years, 10 of them had VSD plus PS, and eight underwent a successful Fontan/BD Glenn repair^[41]^.

**Cardiac transplantation:** Orthotopic heart transplantation, a challenging procedure, may be the only option for patients with refractory heart failure. Although surgical mortality is higher in patients with complex CHD, including CCTGA, who have had multiple previous surgical interventions or whose anatomy makes insertion of a normally formed heart and great vessels less than straightforward, their long-term survival is superior to non-CHD recipients in whom transplantation is a relatively simple procedure^[63]^. Also, a retrospective review of all recipients of donor hearts in the United States of America between 2000 and 2018 who were older than 17 years of age reported better early and long-term outcomes for the patients in whom heart failure secondary of CCTGA was the indication for orthotopic heart transplantation^[64]^.

## CONCLUSION

Every effort should be made for the routine follow-up of adults with CHD. Life-long follow-up is recommended for the great majority of these patients since cure is rare. Adults with complex CHD are at risk of premature death. Particularly the cases of moderate or severe complexity should be seen periodically at the outpatient clinic of ACHD unities by trained experienced physicians and where specific diagnosis and surgical or catheter-based interventions should be done, including non-cardiac procedures, assisted by cardiac anesthetists and intensivists familiar with complex CHD. CCTGA diagnostic criteria are well established. Late diagnosis, particularly in those with no or clinically insignificant lesions, indicate that awareness needs to be improved among physicians dealing with children. The asymptomatic patient should be aware of the potential complications related to the systemic RV^[65]^. RV function is a key prognostic factor for these patients and should be periodically assessed, ideally by CMR. Satisfaction with life and reported health status decline with advancing age indicating that any treatment option should be aimed at improving these parameters^[66]^. Mechanical support in centers with programs that provide such support should be considered as a bridge to transplant or destination therapy (depending on the patient’s age and social situation) for whom heart failure is refractory to medical and any other adjunct therapy, like resynchronization therapy. A recent review focusing on patients with failing systemic RV draw attention for the potential benefits of this technique, but reinforced the need for multicenter investigations in pediatric and adult cohorts with short and long-term assessment^[67]^. Any decisions regarding surgical intervention should take the individual’s anatomy and clinical status into account so that patients are offered the options that offers the least risk and has the highest likelihood of improving the patient’s current clinical status and quality of life. Although there is not yet robust evidence data demonstrating that anatomical surgical correction is beneficial to adult patients with CCTGA, this operation has been successfully performed. New technologies like three-dimensional printing might be useful for surgical planning of the more complex cases^[68]^. The well recognized clinical and morphological variability of individual cases and the appearance of innovative surgical techniques lend themselves to a contemporary update of the multicenter study published two decades ago^[12]^.

**Table:** 

Authors’ Roles & Responsibilities	
FA	Substantial contributions to the conception or design of the work; or the acquisition, analysis, or interpretation of data for the work; drafting the work or revising it critically for important intellectual content; agreement to be accountable for all aspects of the work in ensuring that questions related to the accuracy or integrity of any part of the work are appropriately investigated and resolved; final approval of the version to be published
AMV	Substantial contributions to the conception or design of the work; or the acquisition, analysis, or interpretation of data for the work; drafting the work or revising it critically for important intellectual content; final approval of the version to be published
PHM	Substantial contributions to the conception or design of the work; or the acquisition, analysis, or interpretation of data for the work; drafting the work or revising it critically for important intellectual content; final approval of the version to be published
LGG	Substantial contributions to the conception or design of the work; or the acquisition, analysis, or interpretation of data for the work; final approval of the version to be published
MFBS	Substantial contributions to the conception or design of the work; or the acquisition, analysis, or interpretation of data for the work; drafting the work or revising it critically for important intellectual content; final approval of the version to be published
JMR	Substantial contributions to the conception or design of the work; or the acquisition, analysis, or interpretation of data for the work; final approval of the version to be published
WVAV	Substantial contributions to the conception or design of the work; or the acquisition, analysis, or interpretation of data for the work; final approval of the version to be published
AS	Substantial contributions to the conception or design of the work; or the acquisition, analysis, or interpretation of data for the work; drafting the work or revising it critically for important intellectual content; final approval of the version to be published
